# The interplay between sleep and cancer-related fatigue in breast cancer: A casual and computer-simulated network analysis

**DOI:** 10.1016/j.apjon.2025.100692

**Published:** 2025-03-21

**Authors:** Hongman Li, Ying Xiong, Qihan Zhang, Yufei Lu, Qiaoling Chen, Siqi Wu, Yiguo Deng, Chunmin Yang, M. Tish Knobf, Zengjie Ye

**Affiliations:** a School of Nursing, Guangzhou University of Chinese Medicine, Guangzhou, China; b School of Nursing, Guangzhou Medical University, Guangzhou, China; c Breast Department, Guangdong Provincial Hospital of Chinese Medicine, Guangzhou, China; d School of Nursing, Yale University, Orange, CT, USA

**Keywords:** Sleep quality, Fatigue, Network analysis, Breast neoplasms

## Abstract

**Objective:**

Sleep problems and cancer-related fatigue are common symptoms in women for breast cancer, during and after treatment. Identifying key intervention targets for this symptom cluster may improve patient reported outcomes. This study aimed to explore the relationship between sleep and cancer-related fatigue to identify optimal intervention targets.

**Methods:**

In the “Be Resilient to Breast Cancer” program, self report data were collected on sleep and cancer-related fatigue the Multidimensional Fatigue Symptom Inventory–Short Form and the Pittsburgh Sleep Quality Index. Gaussian network analysis was employed to identify central symptoms and nodes, while a Bayesian network explored their causal relationships. Computer-simulated interventions were used to identify core symptoms as targets for intervention.

**Results:**

General fatigue (Str ​= ​0.95, Bet ​= ​7, Clo ​= ​0.007) was considered the node with the strongest centrality. The daytime dysfunction item on the Pittsburgh sleep quality index had the strongest bridge strength. Core symptoms were identified as targets for intervention by the computer-simulated analysis.

**Conclusions:**

Sleep quality is the strongest predictor of cancer-related fatigue from a casual networking perspective. Sleep latency and daytime dysfunction should be targeted to break the chained symptom interaction between sleep and cancer-related fatigue.

## Introduction

Primary breast cancer is a proliferation of potentially malignant cells within the lumen of the ductal-lobular system.^1^ Cancer-related fatigue (CRF) is one of the most distressing and persistent symptoms reported by women with primary breast cancer.^2^ A survey of 252 Chinese women with breast cancer showed that all participants experienced varying levels of fatigue. Among them, 91.35% reported moderate to severe fatigue.^3^ Cancer-related fatigue refers to persistent physical and emotional exhaustion, often linked to cancer and its treatment.^4^ Unlike common fatigue, cancer-related fatigue does not improve with adequate sleep or rest.^5^

Cancer-related fatigue in patients diagnosed with breast cancer is influenced by various contributing factors, including physiological, psychological, and social aspects.^6^, ^7^, ^8^, ^9^ For example, the expense of treatment can increase financial burden on low-income patients, which in turn may lead to cancer-related fatigue.^10^ In addition, a longitudinal prospective study demonstrated that obese patients with breast cancer experience more cancer-related fatigue than normal weight patients over time.^11^ Furthermore, various studies have shown that fatigue and depression often coexist.^12^^,^^13^

Physiological factors are the most closely associated with cancer-related fatigue, with alterations in sleep being one of the most common factors. Approximately 97% of patients with fatigue experience sleep-related problems, such as difficulty falling asleep, frequent dreams, and poor sleep quality.^14^^,^^15^ These sleep problems can lead to anxiety, irritability, and mental fatigue. Persistent sleep issues may result in endocrine imbalances and weakened immune function, ultimately triggering other health conditions and contributing to the development of cancer-related fatigue.^16^

Notably, sleep and cancer-related fatigue exhibit dynamic and complex interactions^17^ that may significantly impact treatment adherence and quality of life.^4^^,^^7^ Besides, previous studies have shown that sleep issues are often co-occurring with cancer-related fatigue.^18^, ^19^, ^20^ And some studies have primarily analyzed their relationship from a macro (total score of the scale) or overall perspective.^21^, ^22^, ^23^ However, there has been little focus on examining it from a micro (items of the scale) or internal factor perspective. Cancer-related fatigue is interconnected with other symptoms.^24^ Conventional methods, however, may fail to fully capture this intrinsic association between cancer-related fatigue and other symptoms. In contrast, Gaussian network analysis offers a more nuanced approach. It conceptualizes each variable as the result of symptom interactions rather than simply summing symptom scores.^25^ This shift in perspective allows for a deeper understanding of the strength and nature of the relationships between symptoms. However, the causal interpretation of the Gaussian network is limited: it is impossible to tell whether symptom X is more likely to cause or be caused by symptom Y because edges have no direction.^26^ A Bayesian network can overcome these problems and provide deeper insights into determining the direction of causal effects between sleep and cancer-related fatigue.^27^

Furthermore, we need to find intervention targets within the symptoms. Computer-simulated interventions search for optimal intervention targets by altering the activation probabilities of target symptoms.^28^ Unlike traditional clinical interventions, this technique focuses on individual symptoms and shows how these symptoms affect network dynamics.^28^ Additionally, it identifies the symptoms with the greatest impact on overall activation levels. This provides valuable insights for more precise symptom management.^29^

## Theoretical framework

The theory of unpleasant symptoms proposed by Lenz et al. is one of the middle-range theories commonly used in symptom research within nursing.^30^^,^^31^ It aims to assess the physical and psychological responses of patients facing a range of unpleasant symptoms.^30^ This theory explains the correlation between three major components occurring in a patient: (1) the symptoms experienced by the patient (for example, symptoms associated with breast cancer), (2) the factors influencing the symptoms (such as sleep problems and cancer-related fatigue) and (3) the consequences of the symptoms (such as reduce quality of life).^32^ According to the theory, the factors can exist independently or concurrently. However, when multiple factors occur simultaneously, they have a far greater negative impact on the individual than a single factor would have.^33^ Patients diagnosed with breast cancer are often carry a heavy burden from the disease, experiencing unpleasant symptoms such as sleep disturbances and cancer-related fatigue. This, in turn, provides a theoretical foundation for developing more effective treatments and interventions. For patients diagnosed with breast cancer, cancer-related fatigue and sleep issues often interact and converge in the post-surgery phase, during chemotherapy, and as a result of emotional distress and other contributing factors, leading to coexist.^15^ The interplay between these symptoms can exacerbate one another, further complicating treatment and significantly reducing the patient's quality of life.^4^^,^^34^ The theory of unpleasant symptoms helps us gain a deeper understanding of these symptoms and their interrelationships, revealing how they impact the patient's physical and psychological well-being.

Therefore, this study aimed to combine Gaussian network analysis and Bayesian network with computer-simulated interventions to comprehend the relationship between sleep and cancer-related fatigue, ultimately identifying optimal targets for intervention. We hypothesized that:

Hypothesis 1 Core and bridge symptoms are recognized by the Gaussian network analysis.

Hypothesis 2 A Bayesian network explores the direction of causality and offer deeper insights into the most influential symptoms.

Hypothesis 3 Core symptoms as targets for intervention are identified by the computer-simulated analysis.

## Methods

### Participants

Convenience sampling method was used to recruit primary patients undergoing treatment in this study. A sample of 308 female participants with breast cancer from 5 tertiary hospitals in Guangdong, Hunan and Sichuan Provinces were enrolled in “Be Resilient to Breast Cancer (BRBC), from March 2024 to August 2024.^29^^,^^35^^,^^36^ BRBC is a program designed to enhance the mental health of patients diagnosed with breast cancer in mainland China.^37^^,^^38^ They were invited to complete the questionnaire in a quiet room at the hospital, with the first author and the second author providing instructions on how to fill it out. Each participant took approximately 10 minutes to complete the questionnaire independently. The inclusion criteria for the study were: (1) participants willingly agreed to join the study after being approached, (2) a confirmed pathological diagnosis of primary breast cancer, (3) being over 18 years of age, (4) participants were required to be fluent in Chinese with native language proficiency and literacy, (5) currently undergoing chemotherapy, radiotherapy, or endocrine therapy. The exclusion criteria were: (1) a history of diagnosed mental illness, (2) illiteracy or hearing impairments, and (3) recurrent breast cancer or metastatic tumors to the breast.^35^ Initial data inspection found that 17 questionnaires (5.5%) contained a certain amount of missing data. After eliminating these cases, 291 questionnaires were collected in total (response rate ​= ​94.5%).

### Ethical consideration

This study was approved by the ethics committee of Guangdong Hospital of Traditional Chinese Medicine (Approval No. YE2024-109). Before participating in this study, individuals are required to sign an informed consent form to ensure they fully understand the study and voluntarily choose to participate. All personal information in this survey will be kept strictly confidential to protect the patients' rights. After completing the questionnaire, the researcher will inform the patients of their sleep and fatigue levels in person.

### Measures

#### Demographic characteristics

Based on previous studies,^39^ we collected data on clinical,^40^ demographic^35^ and socioeconomic factors.^36^

#### Measurement of cancer-related fatigue

Cancer-related fatigue was measured using the Multidimensional Fatigue Symptom Inventory–Short Form (MFSI-SF),^41^ a 27-item self-report tool designed to evaluate five key dimensions of fatigue: general fatigue (a state of overall fatigue), physical fatigue (for example, arm weakness), emotional fatigue (unpleasant feeling), mental fatigue (for example, difficulty concentrating), and vigor (the body's energy reserves).^42^^,^^43^ The Cronbach's α of the scale in this study is 0.93.

#### Assessment of sleep quality

The Pittsburgh Sleep Quality Index (PSQI), created by Buysse and colleagues,^44^ was utilized to assess sleep quality. The Chinese version has been validated and well adapted by Liu et al.^45^ This index consists of 19 items and is organized into seven domains: sleep quality (one's satisfaction with the sleep experience), sleep latency (the time it takes to transition from wakefulness to sleep), sleep duration (total amount of time asleep), sleep efficiency (the ratio of the amount of total time asleep to the total time in bed), sleep disturbance (condition or issue that disrupts normal sleep patterns), medication use (for example, sleeping pills), and daytime dysfunction (daytime symptoms caused by sleep).^46^^,^^47^ Higher scores on this index indicate poorer subjective sleep quality.^15^ The total score ranges from 0 to 21.^35^ The Cronbach's α of the scale in this study is 0.82.

### Statistical analysis

Descriptive statistics were computed, encompassing frequencies, percentages, means and standard deviations.

First, a Gaussian network analysis was conducted to investigate the relationships between sleep and cancer-related fatigue, and the most core symptoms and bridge symptoms was identified.^48^ High centrality indices were used to determine the core symptoms and bridge symptoms.^49^ The former refers to the highly influential symptoms in the Gaussian network, while the latter refers to symptoms that act as connecting points between different clusters.^50^ Nodes represent symptom items.^51^ To avoid network complexity and overfitting, this study introduces a penalization factor to limit the magnitude of the model's weights, thereby simplifying the model.^52^

Second, a Bayesian network analysis was conducted to identify potential causal relationships between sleep and cancer-related fatigue. Edges along with causal directions were retained.^26^^,^^53^ Directed edges represent a direct or indirect causal link. If symptom X consistently precedes symptom Y, X may be the cause of Y.^27^ The hill-climbing algorithm was used to infer the network structure, and edges along with causal directions that consistently exceeded the threshold across multiple iterations were retained.^54^

Third, computer-simulated interventions were performed to identify the symptom-specific targets. These are the most influential symptoms identified by the computational simulation intervention in sleep and cancer-related fatigue. Computer-simulated interventions offer valuable insights into the intricate dynamics and relationships within symptom networks through advanced network analysis techniques.^55^ Using the NodeIdentifyR algorithm which processes binary data,^56^ we divide the data by assigning “never” to 0 and counting the rest as 1. Two types of interventions were simulated: alleviating intervention and aggravating intervention. This approach helps to uncover which are the most harmful symptoms and potentially informs the development of preventive strategies.^28^

## Results

### Demographic characteristics

Among the 291 participants, the average age was 51.52 years (SD ​= ​11.46), and the average body mass index (BMI) was 23.31 (SD ​= ​3.62). The majority of patients, 226 (77.7%), resided in urban areas, 39.2% were retirees and 65.6% were early stage breast cancer (I and II). Additional demographic information is provided in Table 1.

**Table 1:** Demographic analysis among patients diagnosed with breast cancer.

Variables	*n* (%)
**Age, years (mean ​± ​SD)**	51.52 ​± ​11.46
**Body mass index, kg/m^2^ (mean ​± ​SD)**	23.31 ​± ​3.62
**Educational attainment**	
Secondary school or below	132 (45.4)
High school	68 (23.4)
Associate or bachelor's degree	85 (29.2)
Graduate or postgraduate and above	6 (2.1)
**Employment status**	
Employed	101 (34.7)
Unemployed	76 (26.1)
Retired	114 (39.2)
**Residence**	
City	226 (77.7)
Countryside	65 (22.3)
**Marital status**	
Single	17 (5.8)
Married	248 (85.2)
Divorced	10 (3.4)
Widowed	16 (5.5)
**Annual family income**	
≤ 80,000 RMB (under 11,200 USD)	134 (46.0)
80,000–150,000 RMB (11,200–21,000 USD)	88 (30.2)
150,000–300,000 RMB (21,000–42,000 USD)	52 (17.9)
≥ 300,000 RMB (over 42,000 USD)	17 (5.8)
**Stage of disease**	
Ⅰ	80 (27.5)
Ⅱ	111 (38.1)
Ⅲ	60 (20.6)
Ⅳ	40 (13.7)

∗Guidelines for breast cancer staging system by China Anti-cancer Association (2024 edition).^70^

### Gaussian network analysis

**Fig. 1:**
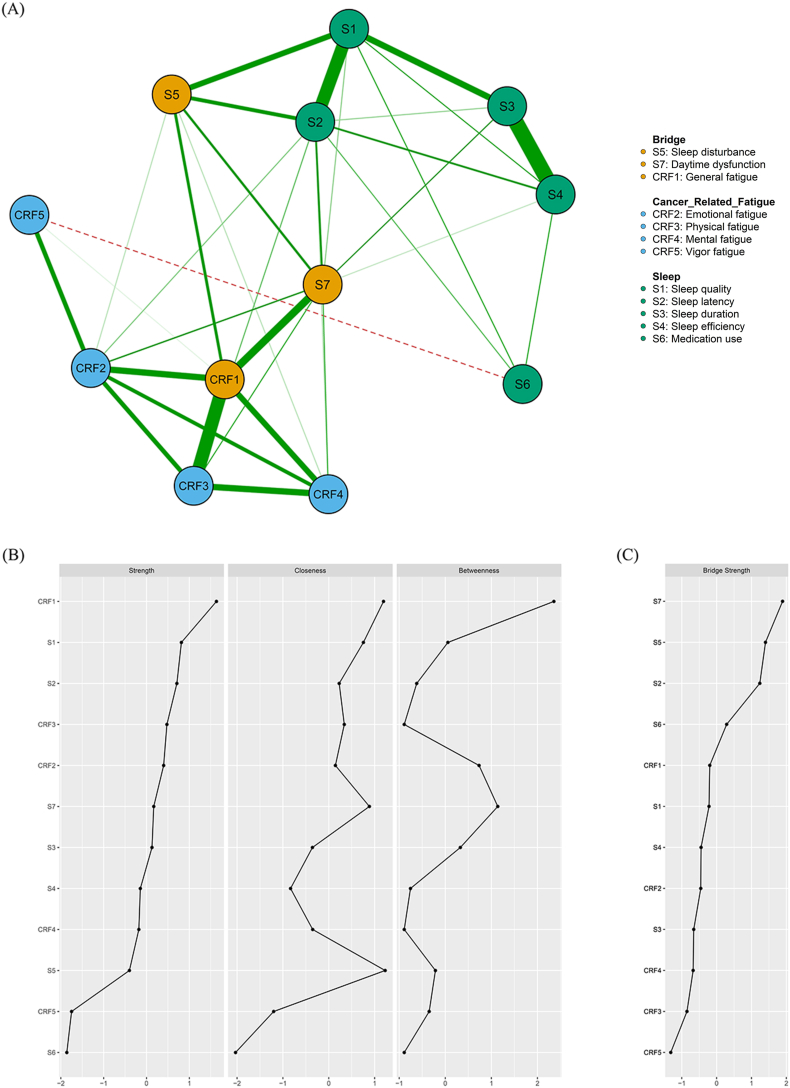
Gaussian network analysis visualization and centrality metrics for sleep and CRF. (A) The depict of Gaussian network structures. (B) Centrality metrics of core symptoms. (C) Centrality metrics of bridge symptoms. CRF, Cancer-related fatigue.

A partial correlation network and Pearson Correlation analysis were used to construct the relationship between sleep and cancer-related fatigue. After removing edges with too little correlation through the penalization factor, the network showed 31 (47.0%) non-zero edges out of a possible 66 edges. In addition, the bridge cores were built to identify the bridge symptoms. The Gaussian network model with other information is shown in Fig. 1 (Gaussian network analysis visualization and centrality metrics for sleep and CRF). Since general fatigue was the strongest node (Str ​= ​0.95, Bet ​= ​7, Clo ​= ​0.007), it was considered the node with the strongest centrality. What's more, daytime dysfunction had the strongest bridge strength and was considered the bridge symptom.

### Bayesian network model

**Fig. 2:**
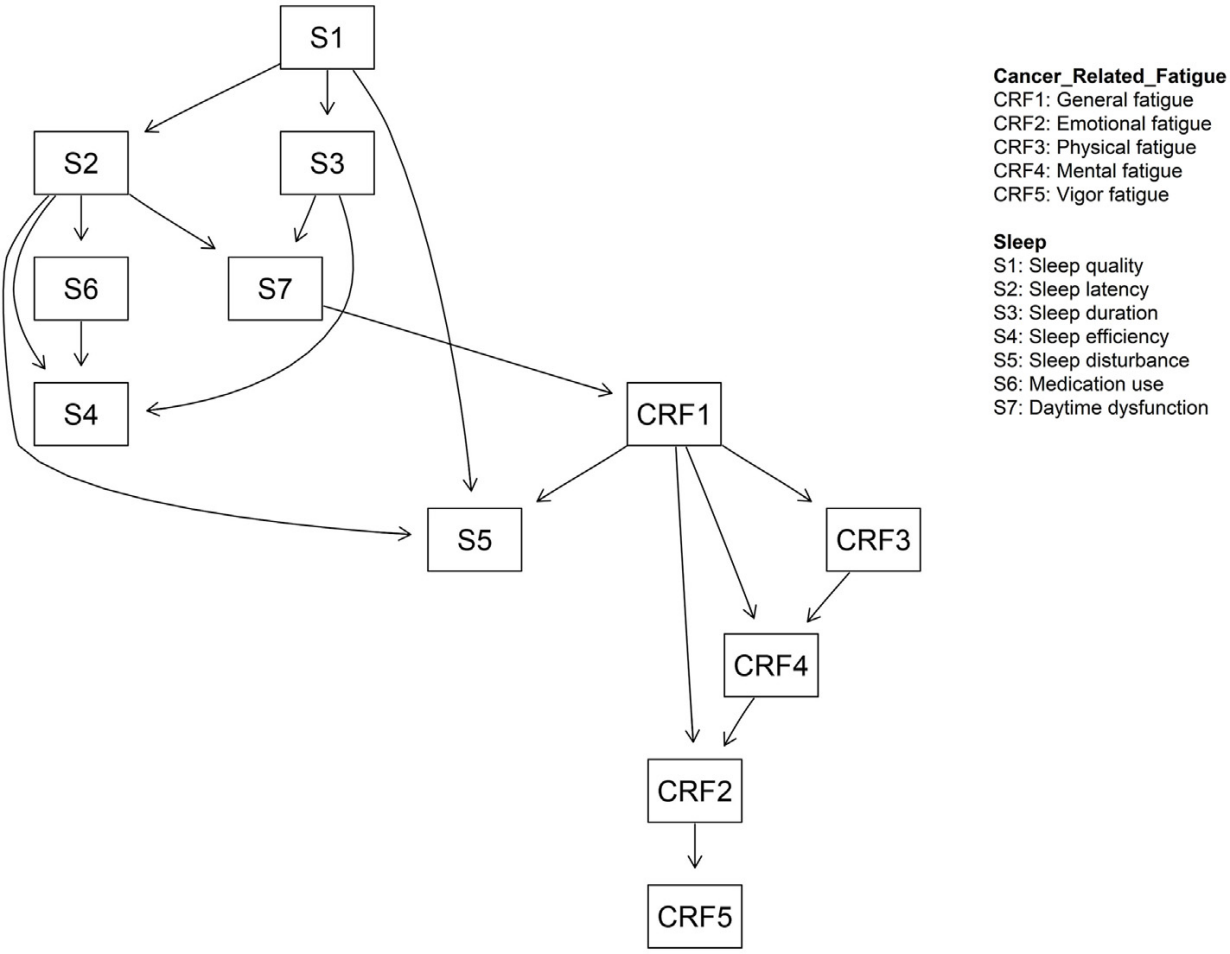
The depict of the Bayesian network model of symptoms. CRF, Cancer-related fatigue.

Directed edges represent potential causal relationships. 17 directed edges were preserved. Due to its position at the top of the figure, sleep quality is identified as the parent node and considered to be the first symptom to appeared. It can directly induce sleep latency, sleep duration, and sleep disturbance, as well as influencing other nodes indirectly. There are only two special causal pathways through that sleep leads to cancer-related fatigue: 1) sleep quality leading to sleep latency, followed by daytime dysfunction, and resulting in general fatigue, and 2) sleep quality leading to sleep duration, followed by daytime dysfunction, and resulting in general fatigue. Furthermore, sleep latency exhibited the most interrelationships within the network and daytime dysfunction was the bridge symptom. Due to their position in the key causal pathways and their unique significance, both sleep latency and daytime dysfunction were identified as key nodes in the Bayesian network. The Bayesian network model with other information is shown in Fig. 2 (the depict of the Bayesian network model of symptoms).

### Effects of computer-simulated interventions

**Fig. 3:**
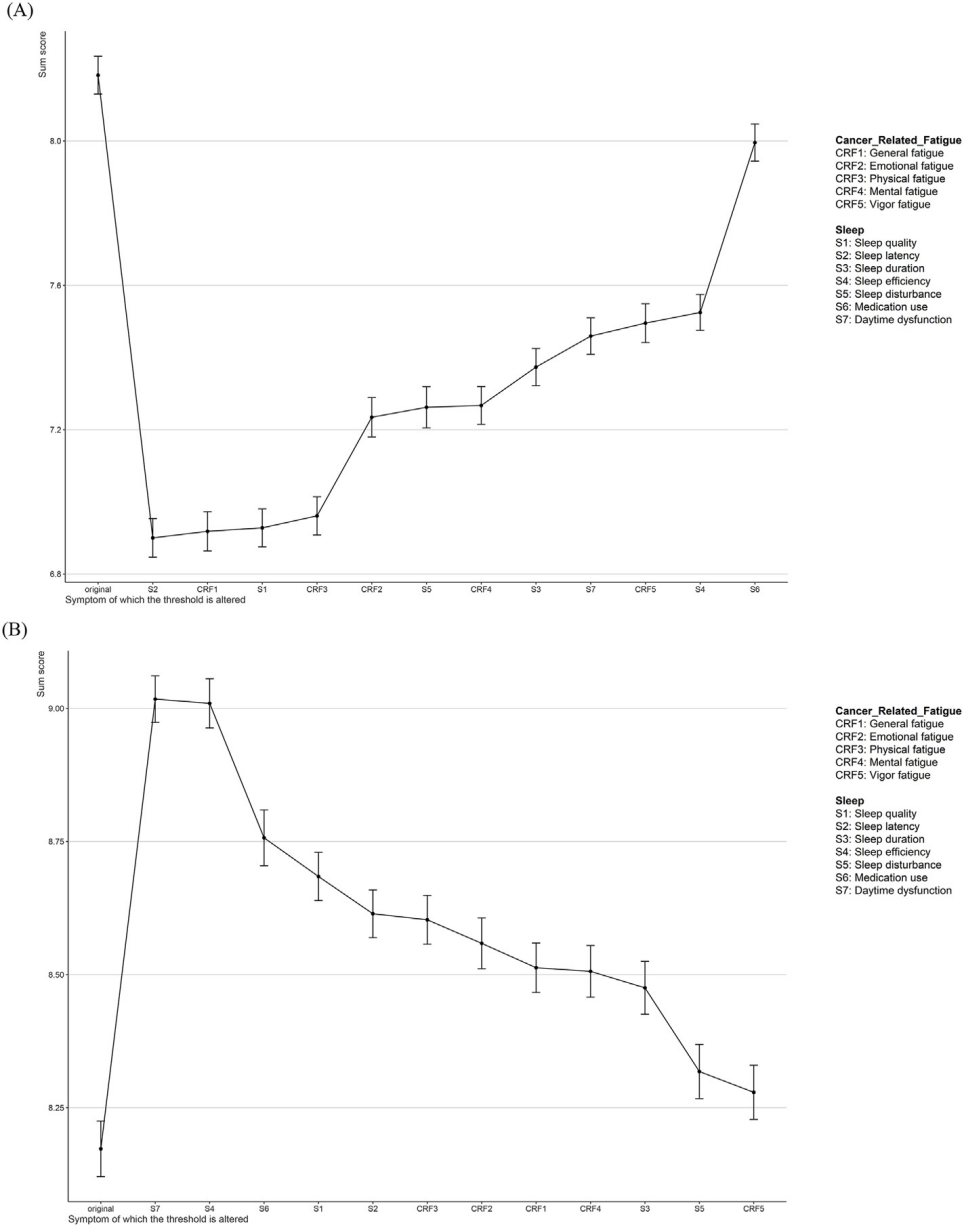
The effect of computer-simulated interventions on the symptoms. (A) Projected Effects of alleviating intervention depicted by sum score of target symptoms. (B) Projected Effects of aggravating intervention depicted by sum score of target symptoms.

The results of the computer-simulated intervention are shown in Fig. 3 (the effect of computer-simulated interventions on the symptoms). The results showed that sleep latency had the lowest sum score during the computerized alleviated intervention, suggesting that sleep latency was the best-alleviated target for this study with sum scores declining from 8.23 to 6.87. This 1.36-point reduction suggested a significant decrease in overall symptom levels within the network. Moreover, general fatigue, as the strongest node, can reduce the overall network score from 8.23 to 6.90; sleep quality, as the parent node in the Bayesian network, can reduce the overall score from 8.23 to 6.92. Both nodes demonstrate significant effectiveness in alleviating the overall symptoms. In addition to this, we also aggravated the intervention by computer simulation, daytime dysfunction significantly increased the overall symptom levels within the network with sum scores rising from 8.10 to 9.03. This increase of 0.93 suggested that daytime dysfunction may be a potential preventive target against symptom aggravation.

## Discussion

The innovation of this study lies in the integration of network analysis and computer simulation interventions. This study showed that general fatigue was the core symptom and daytime dysfunction was considered the bridge symptom. The results of the computer-simulated intervention demonstrated sleep latency was the best-alleviated target and daytime dysfunction may help prevent the worsening of symptoms.

First, the result of the Gaussian network analysis revealed a strong and overwhelmingly positive relationship between sleep and cancer-related fatigue. The central symptom was general fatigue. Most patients diagnosed with breast cancer experience relief from common fatigue following adequate sleep. However, as cancer-related fatigue cannot be fully corrected by sleep, the fatigue continues to affect the patients’ recovery from daytime distress.^47^ General fatigue may lead to symptoms such as difficulty concentrating and delayed responses, while increased physical fatigue reduces activity levels, which can further exacerbate sleep and intensify fatigue.^39^^,^^57^^,^^58^ Notably, daytime dysfunction and general fatigue are strongly correlated (*r* ​= ​0.24), this suggests that women with breast cancer who experience poor sleep tend to have impaired daytime functioning, as evidenced by reduced daytime energy and increased fatigue. Daytime dysfunction has been identified as the most significant bridging symptom, with the greatest likelihood of contributing to other symptoms such as general fatigue. Daytime dysfunction encompasses a range of symptoms arising from insufficient sleep quality or underlying conditions, and is characterized by persistent daytime sleepiness, an excessive preoccupation with sleep, and general dissatisfaction with sleep.^47^^,^^59^ This also suggests that daytime dysfunction is associated with physical fatigue, emotional fatigue, and vigor fatigue, which is consistent with this study.

Second, in the Bayesian network, sleep quality was identified as the key parent node. This suggests that perceived deterioration in sleep quality may be a key characteristic of sleep associated with cancer-related fatigue. This finding aligns with a study on fatigue in cancer patients, which identified sleep quality as a significant predictor of fatigue in this population.^19^^,^^60^ Sleep latency and daytime dysfunction were located at the upper part of the Bayesian network, suggesting that sleep latency and daytime dysfunction were identified as key symptoms contributing to cancer-related fatigue. Sleep latency may interfere with hormonal and metabolic functions, leading to a reduction in growth hormone secretion and an increase in cortisol secretion.^61^ Lower levels of growth hormone contribute to a decline in muscle mass and strength, while elevated cortisol levels impair overall bodily function, which in turn can lead to cancer-related fatigue.^62^^,^^63^ Women with breast cancer undergoing treatments such as chemotherapy and radiotherapy often experience impairments.^29^^,^^64^ These impairments are caused by excessive stimulation of the sympathetic nervous system, which leads to an increased metabolic rate and overconsumption of the body's resources, resulting in physical fatigue.^65^^,^^66^ Additionally, the heightened stress associated with daytime dysfunction can contribute to emotional fatigue in the patients.^67^

Third, the optimal target for preventive care confirmed by computer-simulated interventions was daytime dysfunction. This result is consistent with the finding that daytime dysfunction exhibited the strongest bridge strength, as confirmed by network analysis. Thus, daytime dysfunction has the greatest expected impact on deteriorating sleep combined with cancer-related fatigue symptoms.^68^ Minimizing “sleep latency” has been identified as the most effective target for alleviation of reported sleep problems, suggesting a possible role for interventions like medication, acupuncture, and acupressure.^69^

### Implications for nursing practice and research

In conclusion, sleep quality is a key factor that influences all symptoms and can lead to other symptoms of sleep and cancer-related fatigue. Therefore, special attention should be given to patients diagnosed with breast cancer when they experience a decline in sleep quality. Clinical nurses could carry out preventive interventions for daytime dysfunction, which aggravates intervention target. For patients who have already had sleep problems and cancer-related fatigue, developing an intervention plan on sleep latency (alleviating intervention target) could help reduce the activation of specific symptoms, thereby lowering the overall symptom severity.

### Strengths

This study represents an innovative approach that combines patient symptoms with computer-simulated interventions and offers nurses a more effective method to identify specific targets and develop intervention plans.

### Limitations

This study has certain limitations. The study sample is limited to women with breast cancer from three provinces in China, which may not be representative and carries the risk of selection bias. Future studies should use samples with diverse backgrounds to validate the findings. The intervention targets identified in this study have not been experimentally validated for their effectiveness. Future research should consider randomized controlled trials to assess effects of interventions. Longitudinal designed studies could address changes in sleep and cancer-related fatigue over time and possibly the effect of interventions.

## Conclusions

Sleep quality is the strongest predictor of cancer-related fatigue from a casual networking perspective. By monitoring changes in sleep quality, clinical nurses can identify the potential of cancer-related fatigue in patients diagnosed with breast cancer earlier and actively intervene in sleep latency and daytime dysfunction, thereby reducing the overall severity of symptoms.

## CRediT authorship contribution statement

**Hongman Li:** Conceptualization, Data curation, Methodology, Software, Writing – original draft. **Ying Xiong and Qihan Zhang:** Investigation, Resources, Validation. **Yufei Lu and Qiaoling Chen:** Investigation, Resources. **Siqi Wu and Yiguo Deng: Data curation. Zengjie Ye, M. Tish Knobf and Chunmin Yang:** Supervision, Writing review & editing. All authors read and approved the final manuscript.

## Ethics statement

This study was approved by the ethics committee of Guangdong Hospital of Traditional Chinese Medicine (Approval No. YE2024-109) and was conducted in accordance with the Helsinki Declaration and its later amendments or comparable ethical standards. All participants provided written informed consent.

## Data availability statement

The data that support the findings of this study are available from the corresponding author, ZY, upon reasonable request.

## Declaration of generative AI and AI-assisted technologies in the writing process

No AI tools/services were used during the preparation of this work.

## Funding

This research was funded by grants from the 10.13039/501100001809National Natural Science Foundation of China (Grant No. 72274043, 71904033), the Young Elite Scientists Sponsorship Program by CACM (Grant No. 2021-QNRC2-B08), and Sanming Project of Medicine in Shenzhen (Grant No. SZZYSM202206014). The funders had no role in considering the study design or in the collection, analysis, interpretation of data, writing of the report, or decision to submit the article for publication.

## Declaration of competing interest

The authors declare no conflict of interest. The corresponding author, Prof. Zengjie Ye, is an editorial board member of *Asia–Pacific Journal of Oncology Nursing*. The article was subject to the journal's standard procedures, with peer review handled independently of Prof. Ye and their research groups.
